# High-Sensitivity C-reactive Protein (hs-CRP) as a Biomarker for Predicting Urinary Tract Infection (UTI) Severity and Associated Complications

**DOI:** 10.7759/cureus.91803

**Published:** 2025-09-07

**Authors:** Zafar Ahmad Khan, Ishtiaq Ahmad, Ashraf

**Affiliations:** 1 Urology, Medical Teaching Institution, Mardan Medical Complex, Mardan, PAK; 2 Internal Medicine, Medical Teaching Institution, Mardan Medical Complex, Mardan, PAK; 3 Research and Development, Pro-gene Diagnostics and Research Laboratory, Mardan, PAK

**Keywords:** high-sensitivity c-reactive protein (hs-crp), inflammatory markers, pyelonephritis, urinary tract infection (uti), urosepsis

## Abstract

Background

Urinary tract infections (UTIs) are a common health issue, particularly among women and the elderly, leading to significant morbidity and healthcare burdens. The severity of UTIs can range from uncomplicated cystitis to more severe conditions like pyelonephritis and sepsis, which may cause long-term complications such as renal scarring. High-sensitivity C-reactive protein (hs-CRP), a biomarker of systemic inflammation, has gained attention for its potential role in predicting UTI severity and complications. Understanding the relationship between hs-CRP levels and UTI severity could enhance patient management and treatment strategies.

Objective

This study aims to investigate the relationship between hs-CRP levels and UTI severity, as well as their association with clinical factors such as comorbidities, infection type, and hospital outcomes, with a focus on determining the potential of hs-CRP as a prognostic tool for UTI complications.

Methodology

A cross-sectional observational study was conducted at Mardan Medical Complex from December 2024 to June 2025. A total of 173 UTI patients were included. Participants were categorized into mild, moderate, and severe infection groups based on clinical signs, symptoms, and laboratory results. hs-CRP levels were measured using the Roche Cobas e411 analyzer. Demographic data, comorbidities, infection type, and hospitalization details were collected. Statistical analysis, including ANOVA, Kruskal-Wallis H tests, Mann-Whitney U tests, and decision tree modeling, was used to assess associations between hs-CRP levels and infection severity.

Results

The findings of this study indicate a significant association between elevated hs-CRP levels and the severity of UTIs. The highest hs-CRP concentrations were observed in patients with severe infections, with a mean level of 26.5 mg/L. Moreover, hs-CRP levels were notably correlated with several comorbidities, including hypertension, diabetes, and chronic kidney disease. Specifically, patients with hypertension exhibited a mean hs-CRP level of 24.8 mg/L, while those with diabetes had an average level of 22.6 mg/L. Among individuals with chronic kidney disease, hs-CRP levels were even higher, with a mean of 27.34 mg/L. A decision tree analysis identified distinct hs-CRP thresholds that effectively predicted the severity of infections and the likelihood of complications, such as renal scarring and nephrolithiasis. In terms of pathogen distribution, Escherichia coli was the most frequently identified pathogen, found in 54 patients (31.21%). Notably, antibiotic resistance did not have a significant impact on hs-CRP levels (p=0.972). Nevertheless, hs-CRP was confirmed as a reliable biomarker for assessing infection severity and predicting potential complications. Further analysis revealed a clear gradation in hs-CRP levels corresponding to the severity of infection. Patients with mild infections had a mean hs-CRP level of 15.2 mg/L, those with moderate infections had an average of 21.5 mg/L, and individuals with severe infections presented the highest mean hs-CRP levels of 26.5 mg/L (p<0.001).

Conclusion

Hs-CRP is a reliable biomarker for evaluating UTI severity and predicting complications. Its association with infection severity, comorbidities, and clinical outcomes suggests its potential in clinical decision-making.

## Introduction

Urinary tract infections (UTIs) are prevalent bacterial infections that affect millions globally, with approximately 150 million cases reported each year, posing significant challenges to healthcare systems [1]. They predominantly result from infections of the urinary system by microorganisms, with Escherichia coli being the leading pathogen responsible for the majority of cases [2]. The diversity of UTI presentations ranges from uncomplicated cystitis to life-threatening conditions such as pyelonephritis and urosepsis, underscoring the significant public health concern they represent [3].

The morbidity associated with UTIs can be exacerbated in vulnerable populations, including pregnant women, individuals with underlying comorbidities, and the elderly, who often present atypical symptoms [4,5]. Understanding the inflammatory response in these populations is crucial, as progressions to severe outcomes, such as kidney damage and sepsis, can occur [6]. A critical aspect of managing these infections involves the early identification of infection severity, which can guide clinicians in optimizing treatment regimens and improving patient outcomes.

Compared to the standard CRP assay, the high-sensitivity C-reactive protein (hs-CRP) assay is more sensitive, capable of detecting lower levels of CRP, thereby providing a more precise assessment of a patient’s inflammatory status [7]. The correlation between CRP levels and UTI severity is well-established, with elevated CRP levels serving as a predictor for the increased risk of sepsis [8]. However, there is limited literature on the role of hs-CRP in this context. The utility of the biomarker hs-CRP extends beyond infectious diseases, notably to cardiovascular conditions, where it plays a significant role in assessing systemic inflammation and predicting risks like heart failure [9]. Despite its broad application, the specific role of hs-CRP in diagnosing and understanding the prognosis of UTIs remains an underexplored area. Further research is needed to determine its potential as a reliable biomarker for UTI-related complications and to improve risk stratification in this context.

The inflammatory response measured by hs-CRP levels becomes increasingly relevant in populations that have inherent risk factors for complications. Chronic conditions like diabetes or renal disease are associated with higher hs-CRP levels in response to infections, indicating a more robust inflammatory response that could lead to worse clinical outcomes [10]. For instance, in patients with UTIs, CRP levels are significantly higher in those with UTIs compared to those without [11], highlighting its potential as a prognostic marker for distinguishing between those with and without UTIs. Furthermore, the association of hs-CRP with demographic and clinical factors, including age and comorbidity profiles, provides deeper insight into patient management strategies. Older adults often exhibit elevated CRP levels due to the combined effects of aging and low-grade chronic inflammation [12]. The intricate relationship between these clinical features and hs-CRP levels suggests that clinicians could leverage this biomarker for better risk stratification in patients at high risk for severe UTI complications.

A significant challenge in diagnosing UTIs lies in the early identification of these infections in elderly patients, who often present with atypical symptoms such as confusion or acute changes in mental status, rather than the classic signs of dysuria or frequency. This underscores the difficulty in recognizing UTIs in older adults, as they may not exhibit the typical symptoms seen in younger populations [13]. Reliable biomarkers are crucial to improving diagnostic accuracy. Recent studies suggest that hs-CRP levels are elevated in response to sepsis and septic shock, correlating with the worsening of clinical features indicative of severe infection in elderly patients [14]. This connection enhances the clinical decision-making process, allowing for timely intervention in high-risk populations, such as individuals with multiple comorbid conditions.

The potential of hs-CRP as a prognostic tool extends beyond its current use in assessing cardiovascular disease risk, offering a promising role in diagnosing and predicting the severity of UTIs. Its practical application in both outpatient and inpatient settings makes hs-CRP an accessible, cost-effective tool for healthcare providers, helping to identify patients who may benefit from more aggressive treatment strategies. Given the complexities of UTIs, further research is needed to explore the interactions between inflammatory markers like hs-CRP and clinical outcomes, particularly in relation to antimicrobial resistance patterns, which contribute to increased hospitalizations and morbidity. This approach could improve immediate predictions and inform long-term management and prognosis for recurrent infections, especially in at-risk populations. Incorporating hs-CRP into UTI management may enhance diagnostic accuracy and therapeutic interventions, ultimately improving patient outcomes, with ongoing studies solidifying its role in clinical practice.

## Materials and methods

Study design and setting

This cross-sectional observational study was conducted at the Department of Urology, Mardan Medical Complex, from December 2024 to June 2025. The study aimed to explore the role of hs-CRP levels as a potential predictor of UTI severity and complications. A convenience sampling technique was employed to recruit participants who presented to the hospital with confirmed UTIs during the study period.

Inclusion and exclusion criteria

Adult patients aged 18 years and above, diagnosed with a urinary tract infection confirmed by positive urine culture, scheduled for clinical management at the hospital during the study period, who provided written informed consent, and were clinically stable were included. Patients with underlying conditions affecting CRP metabolism, such as recent use of immunosuppressive drugs or systemic corticosteroids within the past three months, and those with comorbidities like active cancers or autoimmune diseases that could skew hs-CRP levels were excluded.

Sample size calculation

The sample size was determined using G*Power version 3.1 (Heinrich Heine University Düsseldorf, Düsseldorf, Germany) [15], based on a correlational model assessing the relationship between hs-CRP levels and infection severity in UTIs. A moderate effect size (r=0.30) was selected based on prior literature regarding the association of hs-CRP with inflammation and infection outcomes. Using a two-tailed test, with an alpha level of 0.05 and statistical power of 0.80, the minimum required sample size was calculated to be 173 participants. This sample size accounts for possible data loss and ensures sufficient power to detect statistically significant associations between hs-CRP levels and UTI severity and complications.

Demographic and clinical data collection

Demographic and clinical data were obtained from medical records and structured interviews. The following variables were collected: age, sex, body mass index (BMI), blood pressure (systolic and diastolic), smoking history, and the presence of comorbidities (e.g., hypertension, diabetes, and chronic kidney disease). Information on UTI symptoms, including dysuria, frequency, urgency, hematuria, and nocturia, was also gathered. Clinical characteristics such as the presence of renal scarring or nephrolithiasis were also recorded for further analysis.

Microbiological analysis

Urine samples were collected from all participants on admission to confirm the presence of infection. These samples were processed using standard microbiological procedures to isolate pathogens. Antibiotic susceptibility testing was performed on isolated pathogens using the Kirby-Bauer disk diffusion method to identify resistance patterns and guide treatment decisions. The microbial profile was important in categorizing infection types and determining the potential association between specific pathogens and hs-CRP levels.

hs-CRP measurement

Blood samples were collected from each participant for the measurement of hs-CRP levels. Trained phlebotomists drew approximately 5 mL of venous blood from the antecubital vein using standard venipuncture techniques, ensuring sterile conditions to avoid contamination. The blood samples were then transferred to sterile serum separator tubes (SSTs), and participants' serum was separated by allowing the blood to clot at room temperature for 30 minutes, followed by centrifugation at 3,000 rpm for 10 minutes. After centrifugation, the serum was carefully transferred into sterile cryovials for storage. For short-term storage, serum samples were stored at -20°C, while for long-term storage, samples were transferred to -80°C to maintain stability. The hs-CRP levels were then measured using the Roche Cobas e411 analyzer (Roche Diagnostics GmbH, Mannheim, Germany), which utilizes a particle-enhanced turbidimetric immunoassay method. This technique detects changes in turbidity when hs-CRP interacts with specific antibodies, allowing for the precise quantification of even low concentrations of hs-CRP. To ensure accuracy, each batch of measurements was calibrated with known standards of hs-CRP, and internal quality control checks were performed following the manufacturer’s protocol. All samples were analyzed in duplicates to confirm the reliability of the results.

Infection severity classification

Infection severity was classified based on clinical signs and symptoms, laboratory results, and imaging when required. Mild infection included localized symptoms such as dysuria or frequency with no systemic involvement. Moderate infection involved systemic symptoms such as fever, flank pain, or mild leukocytosis. Severe infection was defined by signs of sepsis or systemic inflammatory response syndrome (SIRS), with or without renal involvement such as pyelonephritis [16]. Additionally, complications such as renal scarring, nephrolithiasis, or recurrent infections were considered in determining infection severity.

Statistical analysis

Statistical analyses were performed using SPSS version 29.0 and R (version 4.4.5) (IBM Corp., Armonk, NY, USA) for data visualization. Descriptive statistics were applied to summarize the demographic, clinical, and laboratory data, with continuous variables expressed as means and SD, and categorical variables as frequencies and percentages. For non-parametric data, the Kruskal-Wallis H test was applied, while one-way analysis of variance (ANOVA) was used for parametric data to compare hs-CRP levels across various groups. The Mann-Whitney U test was used for comparing two independent groups. To examine the relationship between hs-CRP levels and clinical biomarkers, feature importance analysis was conducted to evaluate the effect of different biomarkers on hs-CRP levels. Biomarkers were ranked according to their importance scores, highlighting those most closely linked to inflammation. Participants were also categorized into hs-CRP risk groups (low risk, mild risk, moderate risk, and severe risk) based on their hs-CRP levels, and the mean values of biomarkers within these groups were analyzed. Scatter plots were employed to visualize the association between hs-CRP levels and biomarkers. For significant results from ANOVA, Kruskal-Wallis H, and Mann-Whitney U tests, additional analysis was carried out using CDF (cumulative distribution function) and decision tree modeling via the classification and regression trees (CART) algorithm. This approach was used to identify critical hs-CRP thresholds that predict the risk of severe infections or complications. The Gini index was utilized to rank predictor importance in the decision tree model. A p-value of less than 0.05 was considered statistically significant.

Ethical considerations

The study was conducted in accordance with ethical guidelines approved by the Institutional Review Board (IRB) of Mardan Medical Complex, Bacha Khan Medical College. Written informed consent was obtained from all participants before inclusion, ensuring that they understood the study objectives, procedures, and potential risks. All participant data were anonymized and securely stored to maintain confidentiality. The study did not incur additional costs for participants, as laboratory testing was funded by the research department.

## Results

The study included 173 UTI patients, with a mean age of 44.58±18.15 years, and a higher proportion of females (127 patients, 73.41%) compared to males (46 patients, 26.59%). The average systolic blood pressure was 136.88±26.0 mmHg and diastolic blood pressure 90.55±18.4 mmHg, with a mean BMI of 29.76±6.03. Among the participants, 32 patients (18.5%) were smokers, and 141 patients (81.5%) were non-smokers. Regarding comorbidities, 54 patients (27.69%) had no comorbidities, while others had hypertension (40 patients, 20.51%), diabetes (33 patients, 16.92%), and chronic kidney disease (27 patients, 13.85%). Infection severity was categorized as mild (60 patients, 34.68%), moderate (56 patients, 32.37%), and severe (57 patients, 32.95%). Pyelonephritis was the most common infection type (76 patients, 43.93%), followed by cystitis (59 patients, 34.10%). The average onset of infection was 8.17±4.04 days, with hospitalization lasting an average of 4.12±2.0 days. Twenty-seven patients (15.61%) had a history of nephrolithiasis, and 53 patients (30.64%) had urinary tract obstruction (Table 1).

**Table 1:** Demographic, clinical, and infection characteristics of UTI patients Data are presented as mean ± SD for continuous variables and frequency (N) and percentage (%) for categorical variables. UTI, urinary tract infection; BMI, body mass index

Characteristics	Values
Total patients	173 (100%)
Age	44.58±18.15
Gender	
Male	46 (26.59%)
Female	127 (73.41%)
Blood pressure levels	
Systolic blood pressure	136.88±26.0
Diastolic blood pressure	90.55±18.4
BMI	29.76±6.03
Smoking history	
Smoker	32 (18.5%)
Non-smoker	141 (81.5%)
Comorbidities	
None	54 (27.69%)
Hypertension	40 (20.51%)
Diabetes	33 (16.92%)
Chronic kidney disease	27 (13.85%)
Cardiovascular disease	18 (9.23%)
Hepatitis C	10 (5.13%)
Hepatitis B	6 (3.07%)
Asthma	4 (2.31%)
Severity of Infection	
Mild	60 (34.68%)
Moderate	56 (32.37%)
Severe	57 (32.95%)
Type of UTI	
Pyelonephritis	76 (43.93%)
Cystitis	59 (34.10%)
Asymptomatic bacteriuria	15 (8.67%)
Recurrent UTI	14 (8.09%)
Urethritis	9 (5.20%)
Onset of infection days	8.17±4.04
Hospitalization days	4.12±2.0
History of nephrolithiasis	
Yes	27 (15.61%)
No	146 (84.39%)
Urinary tract obstruction	
Yes	53 (30.64%)
No	120 (69.36%)

The study found that the average duration of symptoms in UTI patients was 4.0±3.04 days. The most common symptoms included frequent urination (59 patients, 19.60%), painful urination (53 patients, 17.61%), dysuria (48 patients, 15.95%), urgency (46 patients, 15.28%), nocturia (38 patients, 12.62%), and hematuria (17 patients, 5.56%). Interestingly, 21 patients (12.14%) reported no symptoms. The average duration of hs-CRP elevation was 4.26±3.08 days. Regarding recent surgeries, 11 patients (6.36%) had undergone surgery within the last three months, while 162 patients (93.64%) had not. Additionally, 84 patients (48.55%) experienced urinary retention, and 78 patients (45.09%) had engaged in sexual activity, while 95 patients (54.91%) had not (Table 2).

**Table 2:** Symptom, clinical manifestations, and associated factors in UTI cases Data are presented as mean ± SD for continuous variables and frequency (N) and percentage (%) for categorical variables. UTI, urinary tract infection

Characteristics	Values
Duration of symptoms days	4.0±3.04
Symptoms	
Frequent urination	59 (19.60%)
Painful urination	53 (17.61%)
Dysuria	48 (15.95%)
Urgency	46 (15.28%)
Nocturia	38 (12.62%)
Hematuria	17 (5.56%)
No symptoms	21 (12.14%)
Duration of hs-CRP elevation	4.26±3.08
Recent surgery in the last 3 months	
Yes	11 (6.36%)
No	162 (93.64%)
Urinary retention	
Yes	84 (48.55%)
No	89 (51.45%)
Sexual activity	
Yes	78 (45.09%)
No	95 (54.91%)

The study found that E. coli was the most common pathogen, identified in 54 patients (31.21%), followed by Klebsiella in 52 patients (30.06%) and Proteus in 50 patients (28.90%). Regarding antibiotic treatment, nitrofurantoin was the most frequently used, administered to 38 patients (21.97%), followed by meropenem and ciprofloxacin, each used in 24 patients (13.87%). Nitrofurantoin showed the highest sensitivity in 162 patients (93.64%), followed by meropenem in 114 patients (65.90%) and ceftriaxone in 109 patients (63.01%). Resistance to tetracycline was found in 173 patients (100%), followed by ampicillin in 114 patients (65.90%), cephalexin in 109 patients (63.01%), and amoxicillin in 104 patients (60.12%) (Table 3).

**Table 3:** Microbial profile and antibiotic sensitivity/resistance in UTI infections Data are presented as frequency (N) and percentage (%) for categorical variables. UTI, urinary tract infection

Characteristics	Values
Urine culture results	
Escherichia coli	54 (31.21%)
Klebsiella	52 (30.06%)
Proteus	50 (28.90%)
Enterococcus	6 (3.47%)
Enterobacter	5 (2.89%)
Pseudomonas	3 (1.73%)
Staphylococcus	3 (1.73%)
Antibiotic treatment data	
Nitrofurantoin	38 (21.97%)
Meropenem	24 (13.87%)
Ciprofloxacin	24 (13.87%)
Ceftriaxone	18 (10.40%)
Amikacin	15 (8.67%)
Ampicillin	14 (8.09%)
Sensitive antibiotics	
Nitrofurantoin	162 (93.64%)
Meropenem	114 (65.90%)
Ceftriaxone	109 (63.01%)
Ciprofloxacin	105 (60.69%)
Trimethoprim	105 (60.69%)
Amikacin	62 (35.84%)
Levofloxacin	57 (32.95%)
Ampicillin	56 (32.37%)
Fosfomycin	52 (30.06%)
Vancomycin	06 (3.47%)
Cephalexin	03 (1.73%)
Clindamycin	03 (1.73%)
Resistant antibiotics	
Tetracycline	173 (100.00%)
Ampicillin	114 (65.90%)
Cephalexin	109 (63.01%)
Amoxicillin	104 (60.12%)
Ceftriaxone	52 (30.06%)
Chloramphenicol	50 (28.90%)
Gentamicin	9 (5.20%)
Methicillin	3 (1.73%)

The biomarker profiles of UTI patients revealed various inflammatory and hematological parameters. The mean hs-CRP level was 20.83±11.4, indicating elevated inflammation in these patients. Blood urea nitrogen (BUN) levels had an average of 56.68±60.41, and creatinine levels were 1.39±0.96, reflecting kidney function status. Glycemic control, measured by HbA1c, had an average value of 6.28±2.25. Hematological parameters showed a mean white blood cell count of 11,489.17±5,306.03, with neutrophils making up the largest proportion at 78.3±6.26%. Lymphocytes, monocytes, eosinophils, and basophils had mean values of 18.31±6.33%, 7.27±2.35%, 3.3±1.86%, and 1.65±1.21%, respectively. Platelet count had an average of 276,262.01±71,662.59, and hemoglobin levels averaged 10.85±1.71, indicating moderate anemia in the patient group (Table 4).

**Table 4:** Biomarker profiles in UTI patients: inflammatory and hematological parameters Data are presented as mean ± SD for continuous variables. BUN, blood urea nitrogen; UTI, urinary tract infection; hs-CRP, high-sensitivity C-reactive protein

Characteristics	Values
hs-CRP levels	20.83±11.4
BUN	56.68±60.41
Creatinine levels	1.39±0.96
Glycemic control (HbA1c)	6.28±2.25
White blood cell count	11,489.17±5,306.03
Neutrophils	78.3±6.26
Lymphocytes	18.31±6.33
Monocytes	7.27±2.35
Eosinophils	3.3±1.86
Basophils	1.65±1.21
Platelets	276,262.01±71,662.59
Hemoglobin levels	10.85±1.71

**Figure 1:**
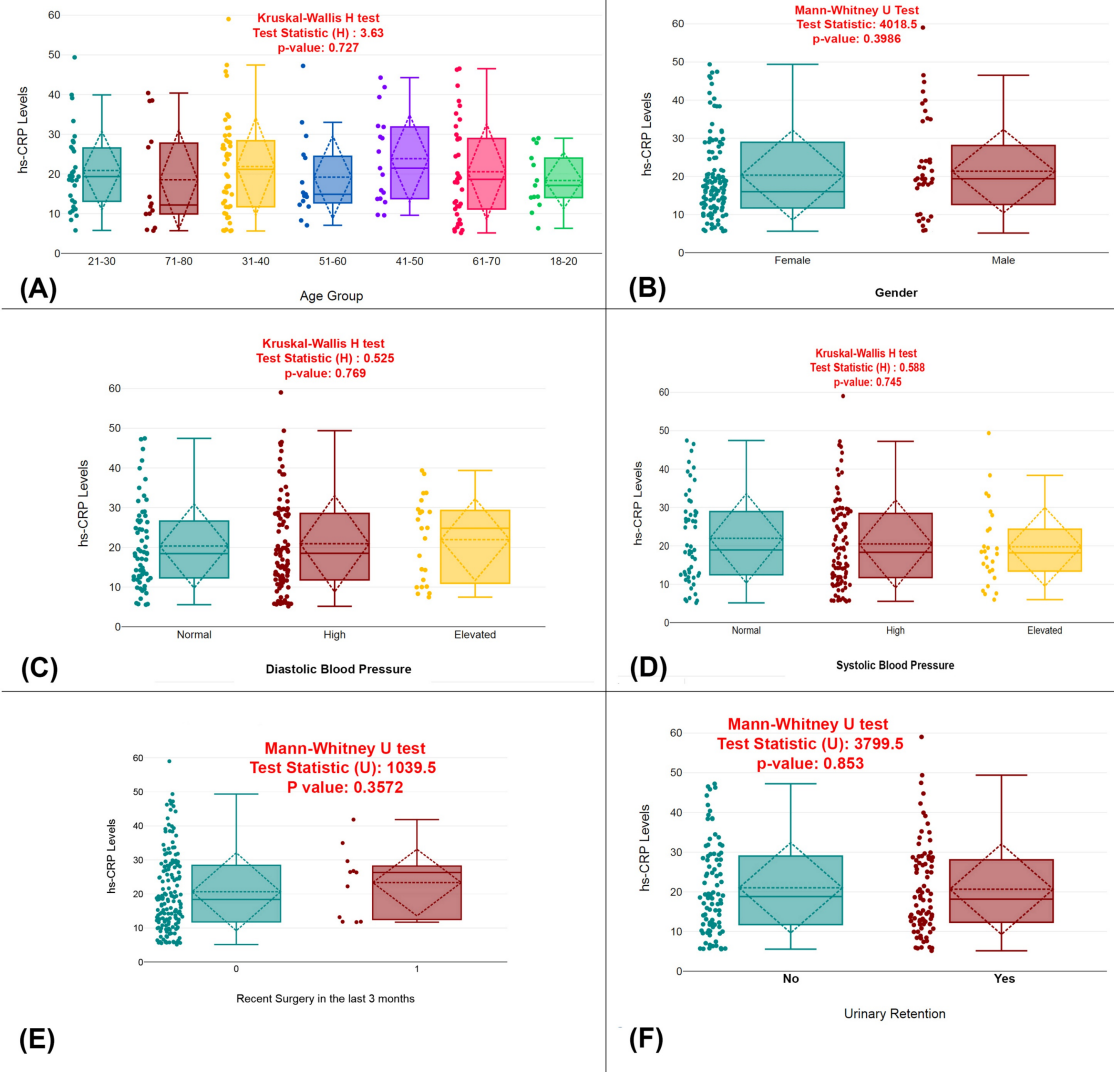
Clinical and demographic factors associated with hs-CRP levels in UTI patients (A) Comparison of hs-CRP levels across different age groups. (B) Comparison of hs-CRP levels between male and female patients. (C) Comparison of hs-CRP levels across diastolic blood pressure categories (normal, high, and elevated). (D) Comparison of hs-CRP levels across systolic blood pressure categories (normal, high, and elevated). (E) Comparison of hs-CRP levels between patients who have had recent surgery in the last three months. (F) Comparison of hs-CRP levels between patients with and without urinary retention. Distribution of hs-CRP levels across various groups with statistical tests (Kruskal-Wallis H and Mann-Whitney U tests); p-value <0.05 is considered statistically significant. UTI, urinary tract infection; hs-CRP, high-sensitivity C-reactive protein

The study analyzed the relationship between various clinical factors and hs-CRP levels in UTI patients. There were no significant differences in hs-CRP levels across different age groups (p=0.727), genders (p=0.3986), or blood pressure categories (both diastolic, p=0.769, and systolic, p=0.745). Additionally, recent surgery in the last three months did not influence hs-CRP levels (p=0.3572), nor did urinary retention (p=0.853). These findings suggest that the examined clinical factors do not significantly affect hs-CRP levels in this cohort of UTI patients (Figure 1).

**Figure 2:**
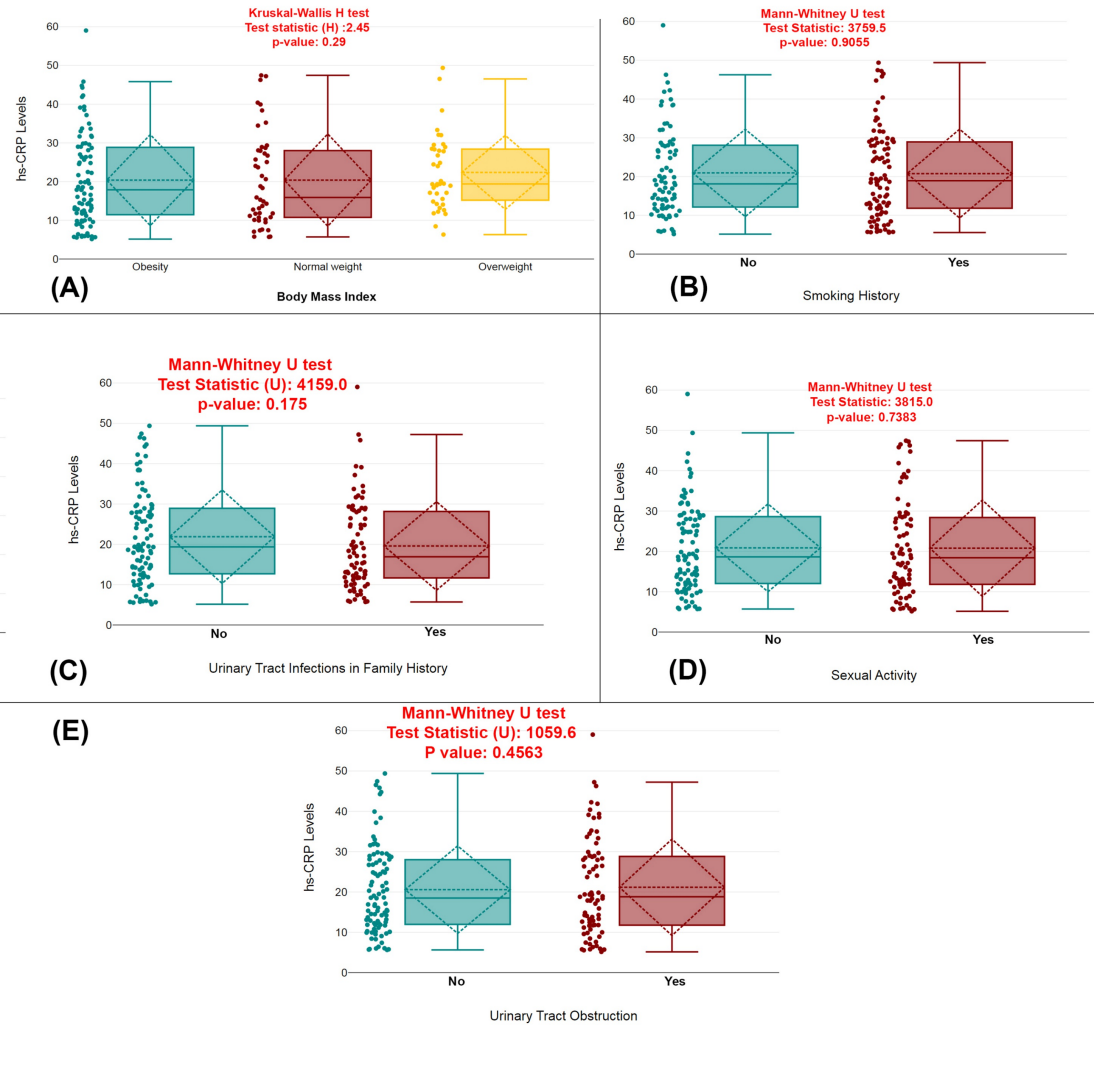
Impact of clinical and demographic factors on hs-CRP levels in UTI patients (A) Comparison of hs-CRP levels across different BMI categories (obesity, normal weight, and overweight). (B) Comparison of hs-CRP levels between smokers and non-smokers. (C) Comparison of hs-CRP levels between patients with and without a family history of UTIs. (D) Comparison of hs-CRP levels between patients with and without a history of sexual activity. (E) Comparison of hs-CRP levels between patients with and without urinary tract obstruction. Distribution of hs-CRP levels across various groups with statistical tests (Kruskal-Wallis H and Mann-Whitney U tests); p-value <0.05 is considered statistically significant. UTI, urinary tract infection; hs-CRP, high-sensitivity C-reactive protein; BMI, body mass index

The analysis reveals that BMI did not significantly affect hs-CRP levels (p=0.29), as determined by the Kruskal-Wallis H test. Similarly, smoking history, family history of UTIs, sexual activity, and urinary tract obstruction also showed no significant impact on hs-CRP levels, with p-values of 0.9055, 0.175, 0.7383, and 0.4563, respectively, as assessed by the Mann-Whitney U test (Figure 2).

**Figure 3:**
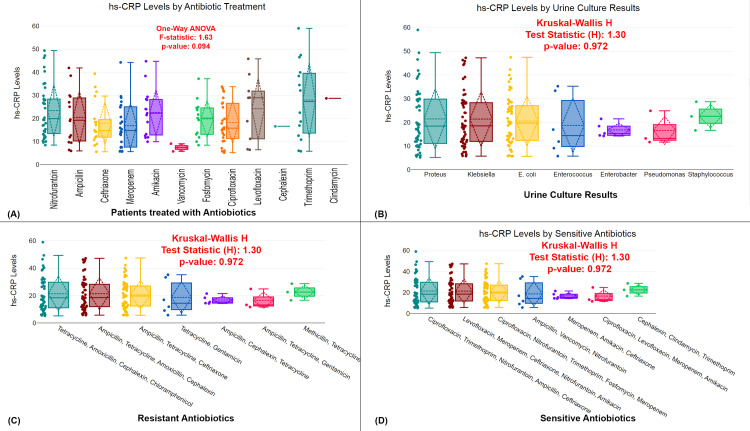
Impact of antibiotic treatment and pathogen resistance on hs-CRP levels in UTI patients (A) Comparison of hs-CRP levels in patients treated with various antibiotics. (D) Comparison of hs-CRP levels by urine culture results, focusing on different pathogens. (C) Comparison of hs-CRP levels in patients treated with antibiotics to which their infections were resistant. (D) Comparison of hs-CRP levels in patients treated with antibiotics to which their infections were sensitive Distribution of hs-CRP levels across various groups with statistical tests (Kruskal-Wallis H and one-way ANOVA); p-value <0.05 is considered statistically significant. UTI, urinary tract infection; hs-CRP, high-sensitivity C-reactive protein

The figure examines the relationship between antibiotic treatment, pathogen culture results, and hs-CRP levels in UTI patients. Figure 3A compares hs-CRP levels across patients treated with different antibiotics (e.g., nitrofurantoin, ampicillin, meropenem, and others), showing no significant differences (p=0.094) based on the type of antibiotic used. Figure 3B explores hs-CRP levels in relation to different urine culture results (Proteus, Klebsiella, E. coli, etc.), and the Kruskal-Wallis H test reveals no significant differences in hs-CRP levels between pathogens (p=0.972). Figure 3C compares hs-CRP levels among patients with resistant infections, showing no significant variation (p=0.972). Similarly, Figure 3D examines hs-CRP levels in patients with infections sensitive to antibiotics, with no significant difference found (p=0.972). Overall, the analysis suggests that neither the type of antibiotic treatment nor pathogen resistance or sensitivity significantly impacts hs-CRP levels in UTI patients (Figure 3).

**Figure 4:**
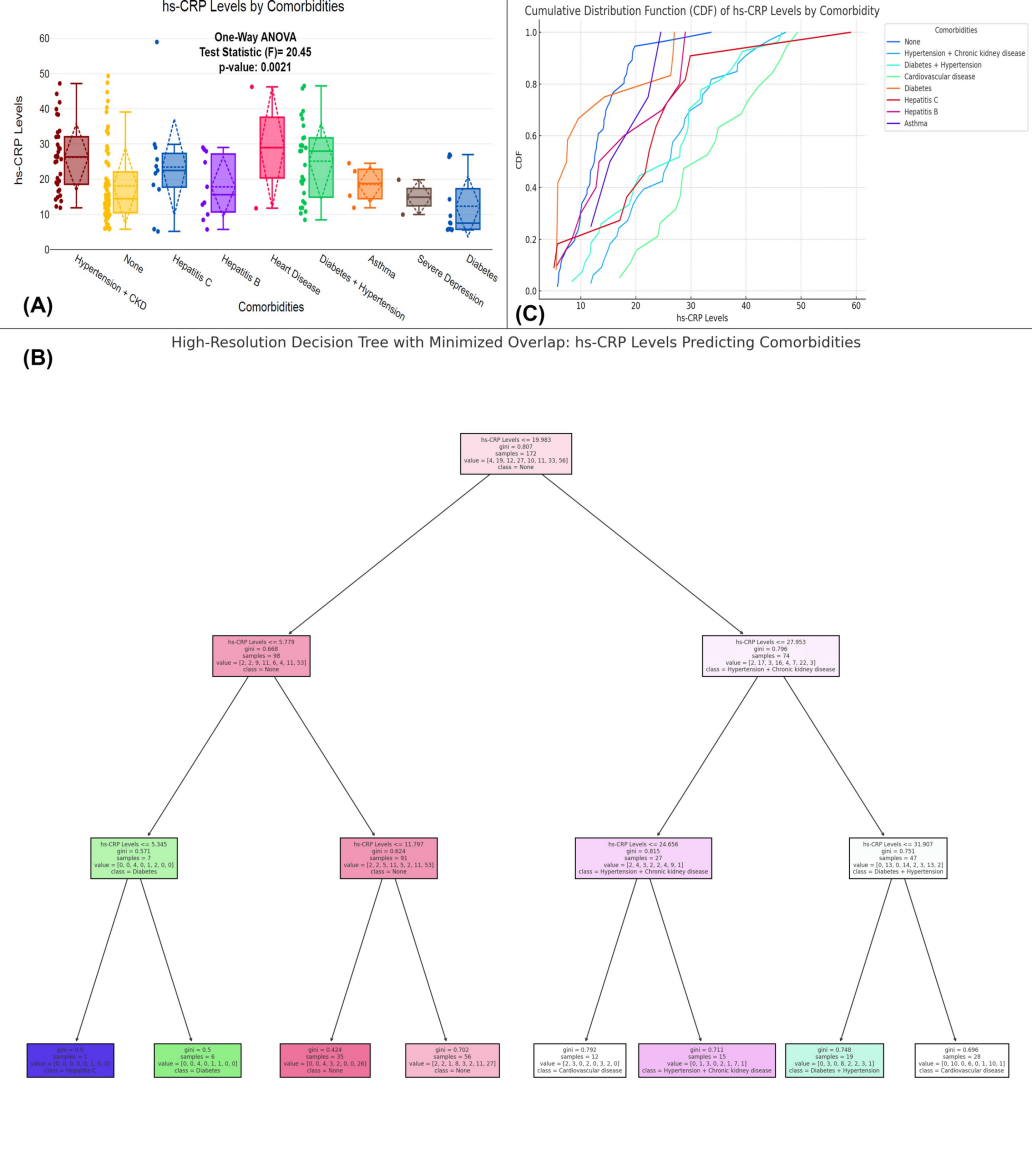
Association between hs-CRP levels and comorbidities in UTI patients (A) Comparison of hs-CRP levels across various comorbidities. (B) A decision tree analysis predicting comorbidities based on hs-CRP levels. (C) A CDF plot comparing hs-CRP levels across different comorbidities Distribution of hs-CRP levels across comorbidities with statistical tests (one-way ANOVA); p-value <0.05 is considered statistically significant. CDF, cumulative distribution function; UTI, urinary tract infection; hs-CRP, high-sensitivity C-reactive protein

The figure explores the relationship between hs-CRP levels and various comorbidities in UTI patients. Figure 4A shows that hs-CRP levels significantly differ across comorbidity groups, with a p-value of 0.0021. The mean hs-CRP levels are highest in patients with hypertension + chronic kidney disease (mean=27.34). The decision tree model predicts comorbidities based on hs-CRP levels by identifying key thresholds for classification (Figure 4B). If hs-CRP ≤9.63 mg/L, the model predicts no comorbidities with a high probability of 94.3%. For levels above this threshold, further splits occur: hs-CRP ≤5.77 mg/L indicates no comorbidity (95.6% probability), while hs-CRP >5.77 mg/L and ≤11.79 mg/L still predict no comorbidity. If hs-CRP >11.79 mg/L, the model predicts hypertension + chronic kidney disease with a 72.5% probability. At hs-CRP >19.63 mg/L, if levels are ≤27.93 mg/L, it predicts hypertension + chronic kidney disease (71.4%), while levels exceeding this threshold predict more severe conditions, including diabetes + hypertension and cardiovascular disease. Figure 4C shows a cumulative distribution function (CDF) plot, where the distribution of hs-CRP levels for hypertension + chronic kidney disease, diabetes, and cardiovascular disease shows a higher shift compared to conditions like hepatitis C, hepatitis B, and asthma.

**Figure 5:**
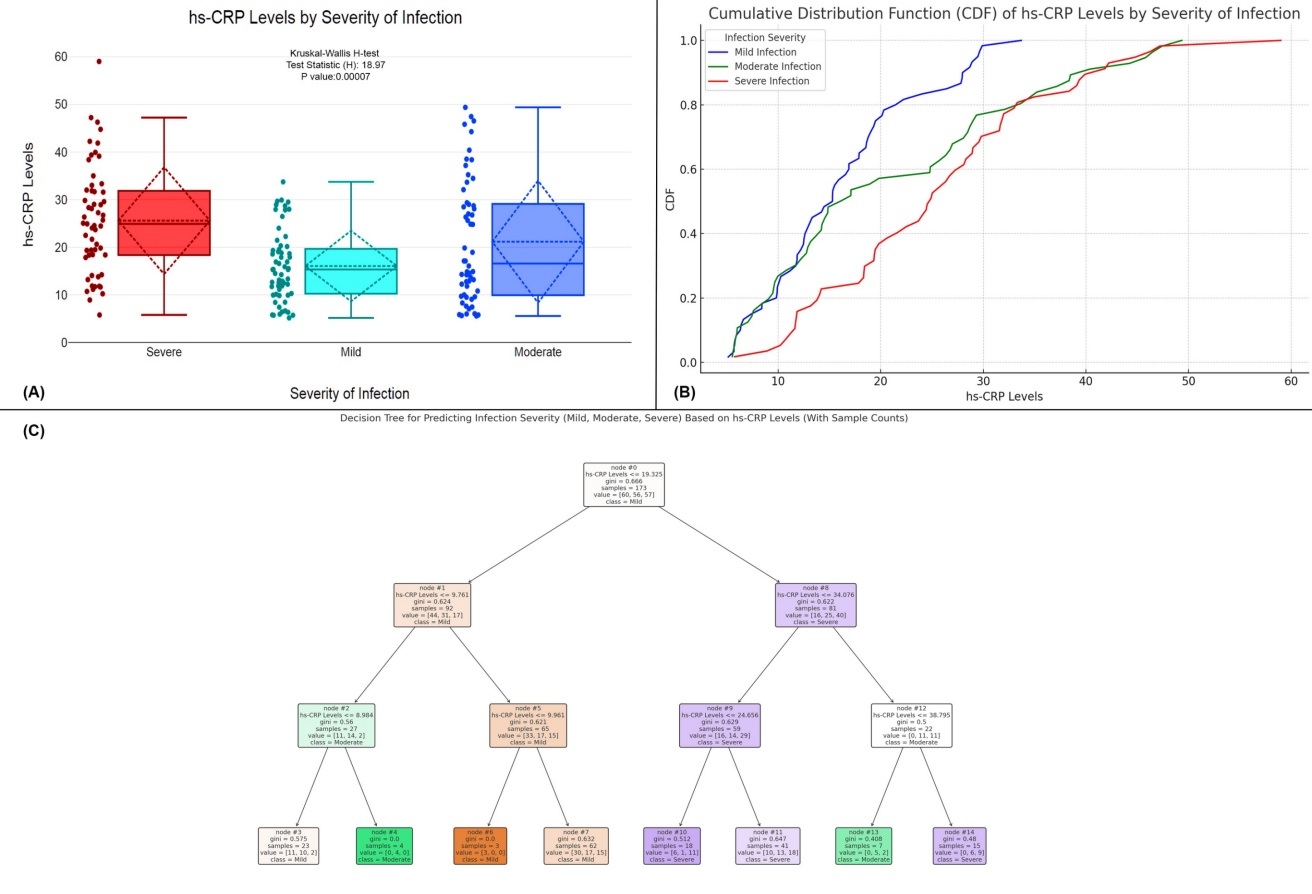
Association of hs-CRP levels with infection severity in UTI patients (A) Comparison of hs-CRP levels across different severity levels of infection. (B) A CDF plot comparing hs-CRP levels across infection severity groups. (C) A decision tree model used to predict infection severity (mild, moderate, and severe) based on hs-CRP levels. Distribution of hs-CRP levels across infection severity with statistical tests (Kruskal-Wallis H); p-value <0.05 is considered statistically significant. CDF, cumulative distribution function; UTI, urinary tract infection; hs-CRP, high-sensitivity C-reactive protein

The figure examines the relationship between hs-CRP levels and the severity of infection in UTI patients. Figure 5A shows that hs-CRP levels significantly differ across severity groups, with severe infections having the highest mean hs-CRP levels (mean=26.5), followed by moderate infections (mean=21.5), and mild infections with the lowest mean hs-CRP levels (mean=15.2). Figure 5B presents a CDF plot, where severe infections have a higher concentration of hs-CRP values compared to mild and moderate infections, which have more spread-out distributions. The decision tree model for predicting infection severity based on hs-CRP levels shows that hs-CRP ≤19.325 mg/L predicts mild infection in 60.35% of cases, while hs-CRP >19.325 mg/L leads to further splits (Figure 5C). For hs-CRP ≤9.761 mg/L, a mild infection is predicted, while hs-CRP >9.761 mg/L suggests a moderate infection. If hs-CRP ≤8.984 mg/L, the infection is classified as moderate in 27 samples. For hs-CRP >9.961 mg/L, the model predicts severe infections if hs-CRP ≤34.076 mg/L, and further divides for hs-CRP >34.076 mg/L, predicting severe infection in 81 samples. Additionally, hs-CRP ≤38.795 mg/L predicts a moderate infection in 22 samples, while higher levels predict moderate and severe infections. This decision tree highlights that higher hs-CRP levels are closely associated with increased infection severity, with severe infections being most predictive at hs-CRP >24.656 mg/L.

**Figure 6:**
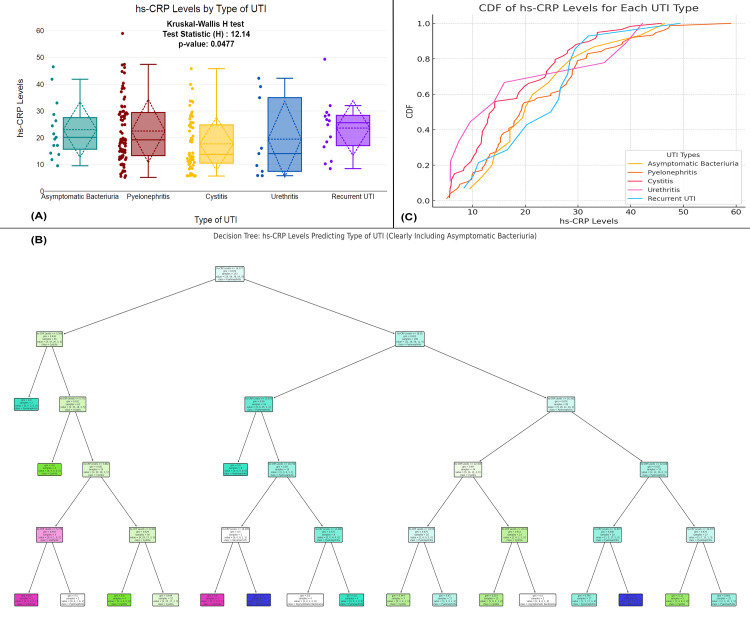
Association between hs-CRP levels and type of UTI in patients (A) Comparison of hs-CRP levels across different UTI types. (B) A decision tree model used to predict the type of UTI based on hs-CRP levels. (C) A CDF plot comparing hs-CRP levels across different UTI types Distribution of hs-CRP levels across types of UTI with statistical tests (Kruskal-Wallis H); p-value <0.05 is considered statistically significant. CDF, cumulative distribution function; UTI, urinary tract infection; hs-CRP, high-sensitivity C-reactive protein

The figure presents an analysis of hs-CRP levels across different types of UTIs. Figure 6A shows a statistically significant difference in hs-CRP levels (p-value=0.00477) among the UTI types, with cystitis having the lowest levels and recurrent UTI the highest. Figure 6C illustrates the CDF for each UTI type, showing distinct distributions with urethritis and pyelonephritis having higher hs-CRP levels compared to others. Figure 6B showcases a decision tree model that predicts UTI type based on hs-CRP levels, clearly distinguishing asymptomatic bacteriuria from the other UTI types. The overall analysis confirms that hs-CRP is a useful biomarker for identifying and differentiating UTI types, particularly distinguishing mild from more severe infections (Figure 6).

**Figure 7:**
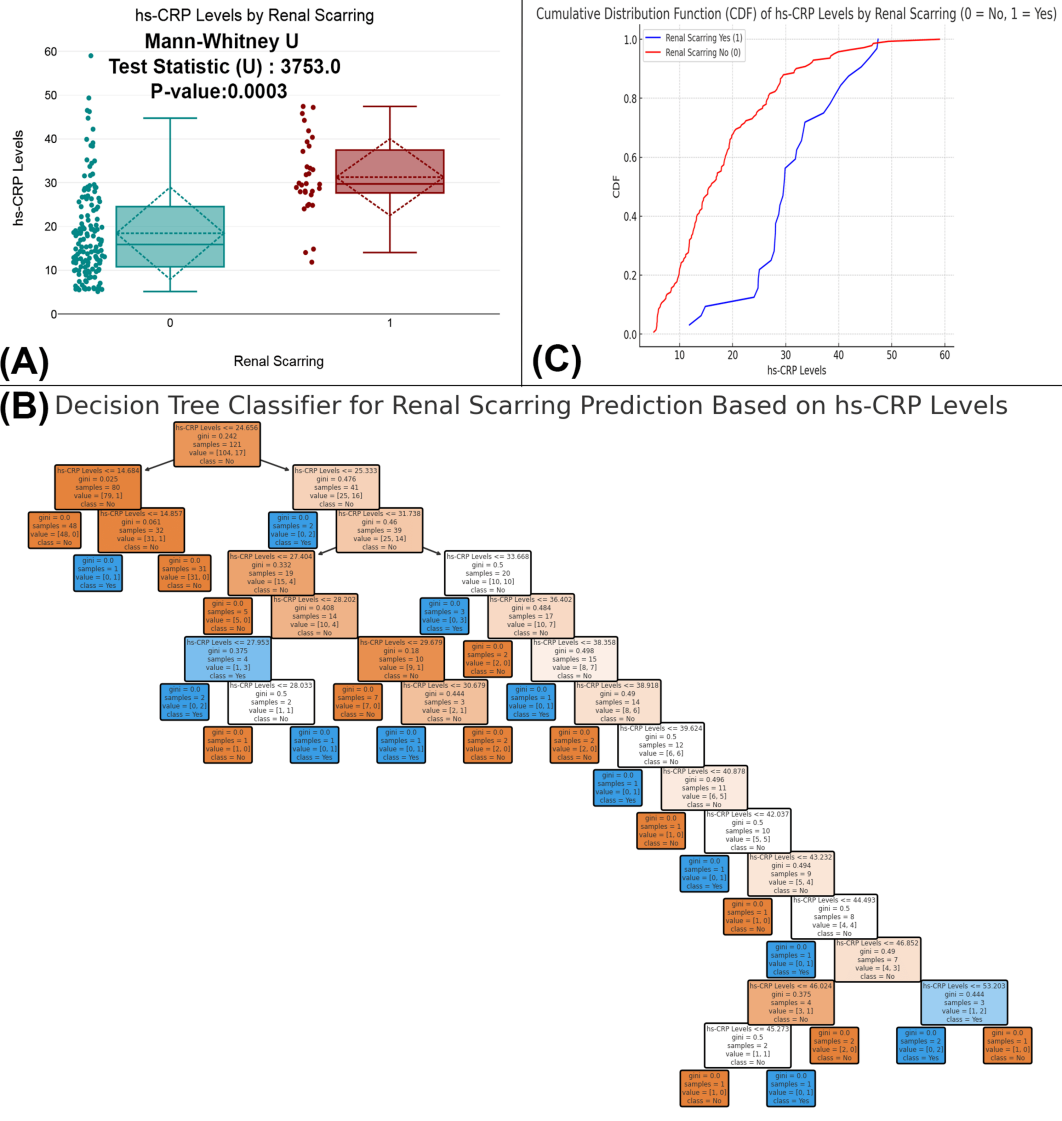
Association of hs-CRP levels with renal scarring in UTI patients (A) Comparison of hs-CRP levels between patients with and without renal scarring. (B) A decision tree model predicting renal scarring based on hs-CRP levels. (C) A CDF plot comparing hs-CRP levels in patients with and without renal scarring. Distribution of hs-CRP levels across renal scarring with statistical tests (Mann-Whitney U); p-value <0.05 is considered statistically significant. CDF, cumulative distribution function; UTI, urinary tract infection; hs-CRP, high-sensitivity C-reactive protein

The figure analyzes the relationship between hs-CRP levels and renal scarring. Figure 7A shows a significant difference in hs-CRP levels between individuals with and without renal scarring (p-value=0.0003), with those having renal scarring exhibiting higher levels. Figure 7C CDF further confirms this trend, as the red curve for individuals with renal scarring lies above the blue curve for those without, indicating higher hs-CRP levels. Figure 7B presents a decision tree model that effectively predicts renal scarring based on hs-CRP levels, showing that elevated hs-CRP levels are associated with a higher likelihood of renal scarring. These findings suggest that hs-CRP is a valuable biomarker for predicting renal scarring (Figure 7).

**Figure 8:**
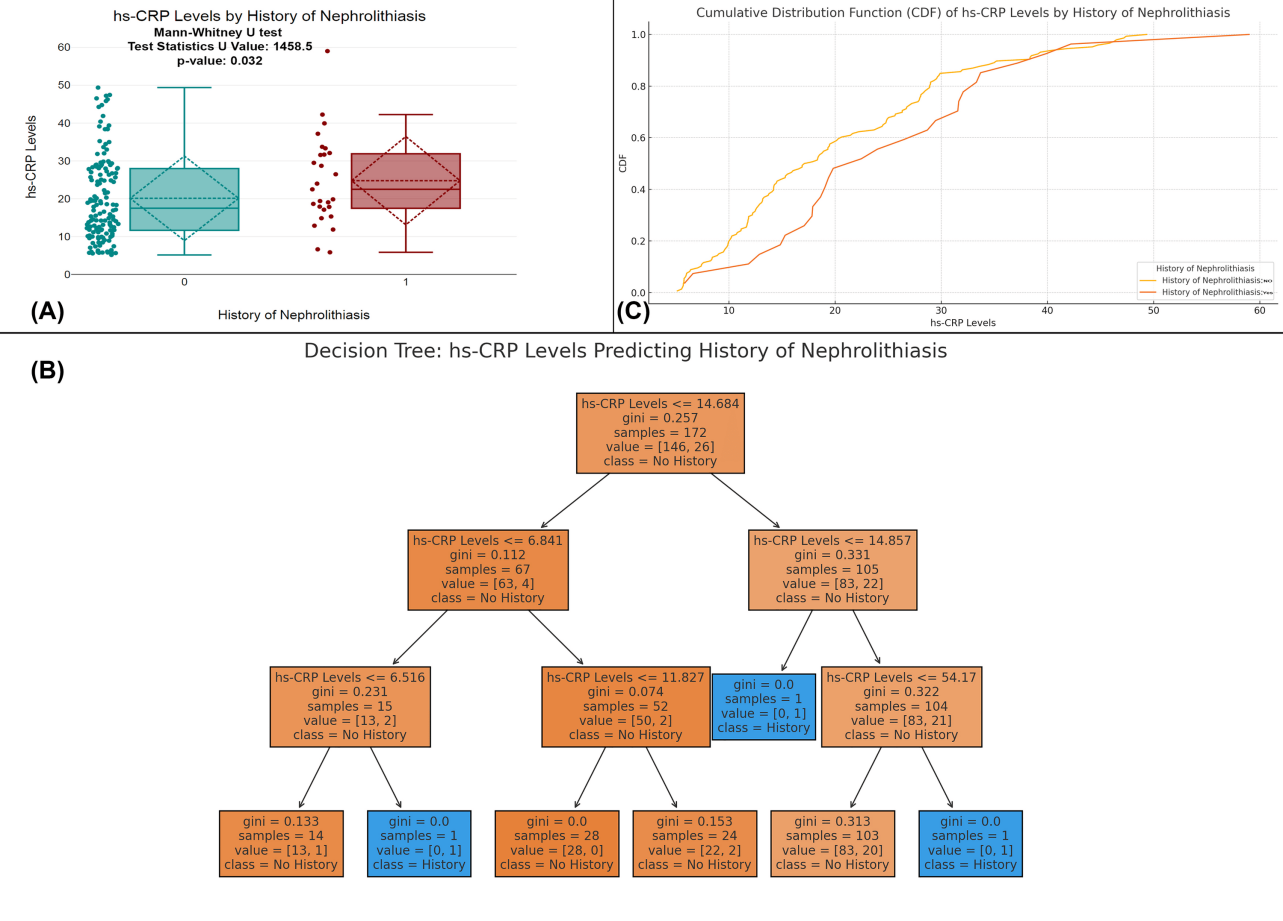
Association of hs-CRP levels with history of nephrolithiasis in UTI patients (A) Comparison of hs-CRP levels between patients with and without a history of nephrolithiasis. (B) A decision tree model for predicting the history of nephrolithiasis based on hs-CRP levels. (C) A CDF plot comparing hs-CRP levels in patients with and without a history of nephrolithiasis. Distribution of hs-CRP levels across the history of nephrolithiasis with statistical tests (Mann-Whitney U); p-value <0.05 is considered statistically significant. CDF, cumulative distribution function; UTI, urinary tract infection; hs-CRP, high-sensitivity C-reactive protein

The figure examines the relationship between hs-CRP levels and a history of nephrolithiasis (kidney stones). Figure 8A shows a significant difference in hs-CRP levels between individuals with and without a history of nephrolithiasis (p-value=0.032), with those having a history displaying higher hs-CRP levels. Figure 8C CDF further supports this, as the curve for individuals with a history of nephrolithiasis lies above the curve for those without. Figure 8B presents a decision tree model, which uses hs-CRP levels to predict a history of nephrolithiasis, showing specific thresholds where higher levels of hs-CRP are associated with the presence of the condition. Overall, these findings suggest that hs-CRP is a useful biomarker for predicting a history of nephrolithiasis (Figure 8).

**Figure 9:**
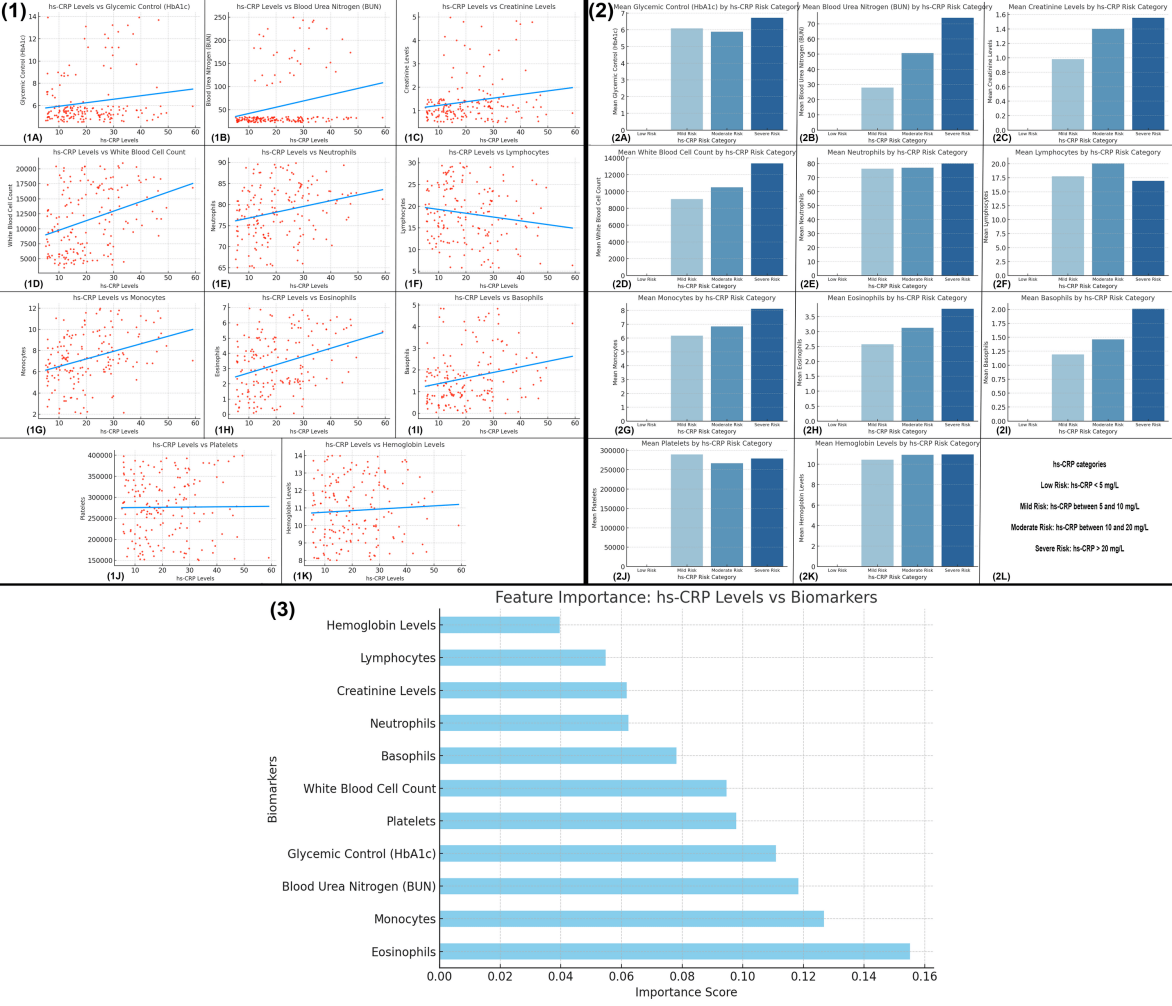
Biomarker profiles and their association with hs-CRP levels in UTI patients UTI, urinary tract infection; hs-CRP, high-sensitivity C-reactive protein

The figure examines the relationship between hs-CRP levels and various biomarkers in UTI patients, revealing several important findings. Figure 9 shows that glycemic control (HbA1c), kidney function markers (BUN and creatinine), and inflammatory markers (WBC and neutrophils) have moderate positive correlations with hs-CRP levels, indicating that higher levels of these biomarkers are associated with elevated hs-CRP. In contrast, lymphocytes show a slight negative correlation with hs-CRP, while other biomarkers like monocytes, eosinophils, and basophils exhibit weaker correlations. Hemoglobin levels are negatively correlated with hs-CRP, suggesting that lower hemoglobin levels are linked to higher hs-CRP levels. Figure 9 highlights that as hs-CRP levels increase, biomarkers such as HbA1c, creatinine, WBC, neutrophils, and platelets show a consistent increase, while lymphocytes and hemoglobin levels decrease. Figure 9 shows feature importance scores for predicting hs-CRP levels, with hemoglobin, lymphocytes, and creatinine being the most influential biomarkers, followed by neutrophils and basophils. Overall, the analysis underscores the significant role of inflammatory markers, kidney function indicators, and glycemic control in predicting hs-CRP levels in UTI patients.

**Figure 10:**
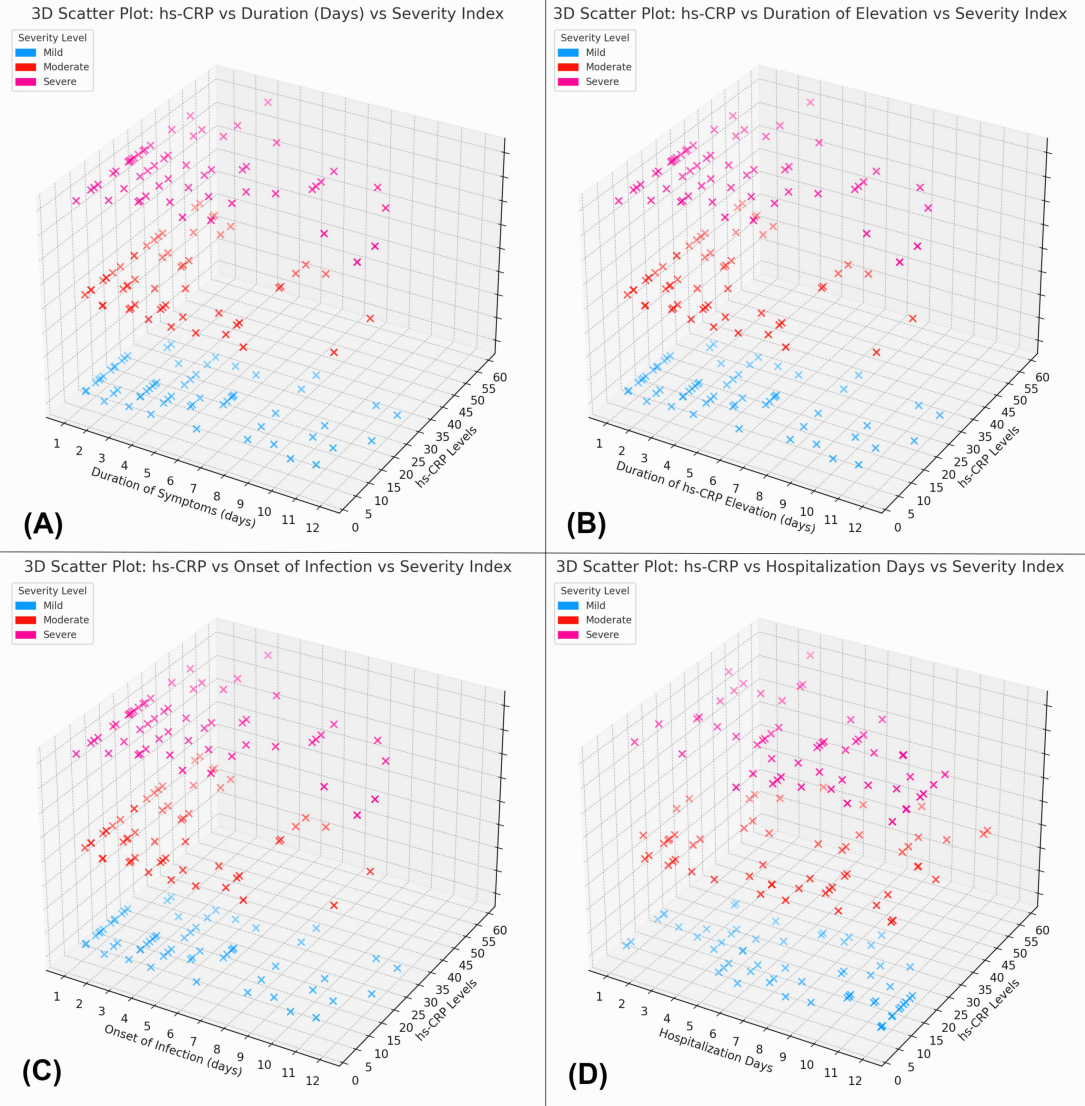
Relationship between hs-CRP levels, duration of symptoms, and severity index in UTI patients (A) 3D scatter plot illustrating the relationship between hs-CRP levels, duration of symptoms (in days), and the severity index. (B) 3D scatter plot illustrating the relationship between hs-CRP levels, the duration of hs-CRP elevation (in days), and the severity index. (C) 3D scatter plot showing the relationship between hs-CRP levels, the onset of infection (in days), and the severity index. (D) 3D scatter plot illustrating the relationship between hs-CRP levels, hospitalization days, and the severity index. UTI, urinary tract infection; hs-CRP, high-sensitivity C-reactive protein

The figure presents 3D scatter plots analyzing the relationship between hs-CRP levels and clinical variables across different UTI severity levels. Figure 10A shows that longer symptom duration is associated with higher hs-CRP levels, particularly in severe cases. Figure 10B indicates that longer durations of hs-CRP elevation correlate with higher hs-CRP levels, with severe cases showing the highest levels. Figure 10C demonstrates that a later onset of infection is linked to higher hs-CRP levels, especially in severe cases. Finally, Figure 10D reveals that longer hospitalization is associated with elevated hs-CRP levels, with the most severe cases exhibiting both longer hospital stays and higher hs-CRP levels. Overall, the analysis underscores the relationship between increased hs-CRP levels and more severe UTI outcomes, reflecting the severity of infection and its clinical course.

## Discussion

The role of hs-CRP in UTIs has gained considerable interest in clinical research. This study examined hs-CRP levels in 173 UTI patients, exploring its potential as a biomarker for assessing infection severity, predicting complications, and identifying patients at risk for adverse outcomes. Compared to existing literature, there is limited data directly addressing this topic. The study's key findings revealed a predominance of females in the cohort, consistent with established epidemiological trends. Women are especially prone to UTIs due to anatomical factors, such as a shorter urethra, which allows bacteria to more easily ascend from the perineum to the bladder [17]. Hormonal fluctuations, sexual activity, and certain contraceptive methods further increase this susceptibility, contributing to a higher recurrence rate [18].

Further analysis of comorbidities revealed a substantial prevalence of conditions such as hypertension, diabetes, and chronic kidney disease among the participants. This aligns with prior studies that have identified these comorbidities as significant risk factors for complicated UTIs. For instance, diabetes is well-documented to compromise immune function, increasing susceptibility to infections and exacerbating the severity of UTIs [19]. Hypertension, often associated with kidney damage [20], raises the risk for pyelonephritis and recurrent infections. Existing studies corroborate these findings, highlighting the significance of underlying health conditions in influencing infection pathways and outcomes.

The classification of UTI severity into mild, moderate, and severe categories illustrates the heterogeneity of clinical presentations. The study reported a significant incidence of pyelonephritis, found in 43.93% of cases, which is well-supported by existing research that recognizes this condition as a precipitant of serious complications, including renal abscesses or systemic sepsis if left untreated [21,22]. Additionally, common forms of UTIs identified included cystitis and recurrent infections [23,24], underscoring the necessity for ongoing vigilance and management strategies to mitigate patient morbidity associated with these conditions.

The liver produces hs-CRP in response to inflammatory cytokines, particularly interleukin-6, reflecting a strong immune response linked to more severe inflammatory profiles [25]. This process underscores hs-CRP's potential role in assessing infection severity, informing treatment decisions, and predicting complications. Importantly, the study revealed a significant correlation between elevated hs-CRP levels and the severity of UTI infection. Notably, patients suffering from severe infections exhibited markedly higher hs-CRP levels compared to those with mild forms of the disease. This finding supports the premise that hs-CRP serves as a sensitive marker of systemic inflammation during the course of infection [26].

The association of prolonged symptoms and extended hospital stays with increased hs-CRP levels reflects a previous study asserting that chronic inflammation is linked to poorer clinical outcomes in UTIs [27]. Elevated hs-CRP signifies a likely severe infection requiring a more pronounced immune response; however, chronic inflammation may concurrently predispose patients to adverse outcomes, such as renal damage or septic complications [28]. These insights reaffirm the notion that hs-CRP can function not only diagnostically but also prognostically, guiding clinical decision-making to improve patient management.

Contrary to expectations, no significant differences in hs-CRP levels were observed across various antibiotic treatments, pathogen types, or antibiotic resistance patterns, suggesting that CRP primarily reflects systemic inflammation [29] rather than being influenced by specific microbial agents or their susceptibility to antibiotics. This finding highlights the nonspecific nature of hs-CRP as an inflammatory marker and reaffirms previous research that CRP levels gauge general inflammation rather than the specifics of infection. Therefore, while hs-CRP is valuable in assessing UTI severity, it should not replace comprehensive microbiological evaluations and careful antibiotic selection. The lack of correlation between antibiotic resistance and hs-CRP levels calls attention to the complex relationship between persistent infections, host responses, and treatment efficacy, underscoring the need for clinical judgment to guide treatment decisions alongside biomarker assessments.

The study also highlighted significant associations between hs-CRP levels and complications such as renal scarring and nephrolithiasis. Patients presenting with renal scarring exhibited notably elevated hs-CRP levels, reinforcing the significance of timely intervention in UTI management to avert long-term kidney damage. This underscores the broader clinical implications of UTI management, where early diagnosis and comprehensive treatment are paramount in preventing potentially life-altering renal complications [30].

The study's decision tree analysis identified specific hs-CRP thresholds predictive of underlying comorbidities. For instance, hs-CRP levels exceeding 22.73 mg/L were consistently associated with diabetes, while levels above 19.73 mg/L correlated with hypertension. Given the accessibility and cost-effectiveness of hs-CRP testing, its incorporation into standard clinical practice could yield significant improvements in patient outcomes through early detection and intervention strategies.

Limitations of the study

Despite the significant findings of this study, there are several limitations to consider. First, the cross-sectional design of the study limits the ability to establish causal relationships between hs-CRP levels and infection severity or complications. A longitudinal study design, which follows patients over time, would provide more robust data on how hs-CRP levels change throughout the course of infection and treatment, as well as how these changes correlate with clinical outcomes. Second, while hs-CRP was used as a marker of inflammation, it is a nonspecific biomarker. Elevated hs-CRP levels can be seen in a wide range of inflammatory conditions, including infections, autoimmune diseases, and even cardiovascular conditions. Therefore, while hs-CRP is useful for assessing inflammation, it may not provide detailed insights into the underlying etiology of infection or the specific mechanisms driving inflammation. Third, convenience sampling was employed due to resource constraints, which introduces selection bias and limits the generalizability of the findings to the broader population. This method may affect the external validity of the conclusions, as it is inherently limited by the sample's representativeness. Future studies using more robust sampling methods, such as random sampling, would be valuable for confirming the applicability of these results. Finally, the study was conducted at a single tertiary care center, which may limit the generalizability of the findings to other populations or healthcare settings. Future studies involving multiple centers and diverse patient populations would help confirm the applicability of these results in broader clinical contexts.

Clinical implications

The findings of this study have several important clinical implications. Elevated hs-CRP levels were strongly associated with the severity of UTIs, making it a potentially valuable biomarker for assessing infection severity and predicting the risk of complications. Early identification of patients with elevated hs-CRP levels could prompt more aggressive monitoring and treatment, potentially improving patient outcomes by preventing the progression of infection to more severe forms such as pyelonephritis or sepsis. In particular, hs-CRP could be used as a tool to identify high-risk patients who may benefit from closer follow-up or more intensive treatment. For example, patients with elevated hs-CRP levels and comorbidities such as diabetes or hypertension may be at higher risk for severe infections or complications, and therefore may require more aggressive management strategies. Additionally, hs-CRP could serve as a useful marker for monitoring the effectiveness of treatment, providing clinicians with real-time information on the patient’s inflammatory response and the likelihood of infection resolution.

## Conclusions

This study provides valuable insights into the role of hs-CRP as a biomarker for assessing the severity of UTIs and predicting complications. Elevated hs-CRP levels were associated with more severe infections, longer symptom durations, and comorbidities such as diabetes and hypertension. Furthermore, hs-CRP levels were predictive of the development of renal scarring and nephrolithiasis, highlighting its potential utility in identifying patients at risk for long-term complications. However, further research, particularly longitudinal studies, is needed to fully understand the dynamics of hs-CRP in UTI patients and its potential role in guiding clinical decision-making.

## References

[1] Turyatunga G (2023). The prevalence of bacterial pathogens associated with urinary tract infection (UTI) among patients attending Kam Medical Consult Clinic, Uganda. J Health Res Africa.

[2] Waqas M, Khan Z, Ahmad S (2024). Antibiotic sensitivity and resistance patterns of uropathogens in Peshawar, Pakistan. J Health Res Rep.

[3] Craven BC, Alavinia SM, Gajewski JB (2019). Conception and development of urinary tract infection indicators to advance the quality of spinal cord injury rehabilitation: SCI-High Project. J Spinal Cord Med.

[4] Obeng-Nkrumah N, Korang-Labi A, Kwao P (2024). Extended-spectrum beta-lactamase-producing Enterobacterales in human health: experience from the tricycle project, Ghana. PLoS One.

[5] FitzGerald M, Mueller E, Brubaker L, Dalaza L, Abraham T, Schreckenberger P (2008). Chronic lower urinary symptoms do not predict bacteriuria. Int J Gynaecol Obstet.

[6] Mhana S, Aljanaby A (2023). Bacteriological and immunological study of kidney failure patients infected with urinary tract infections. E3S Web Conf.

[7] Jiang Z, Jiang H, Zhu X, Zhao D, Su F (2024). The relationship between high-sensitivity C-reactive protein and gallstones: a cross-sectional analysis. Front Med (Lausanne).

[8] Bharath M, Hiremath R, Basu A (2017). Role of procalcitonin and C-reactive protein in urinary tract infection diagnosis in adults. Int J Adv Med.

[9] Fatima Z, Shakeel R, Chaudhry SA (2025). The relation between C-reactive protein (CRP) and risk of incident heart failure in patients with cardiovascular disease: a narrative review. Ann Med Surg (Lond).

[10] Lucci C, Cosentino N, Genovese S (2020). Prognostic impact of admission high-sensitivity C-reactive protein in acute myocardial infarction patients with and without diabetes mellitus. Cardiovasc Diabetol.

[11] Leventoğlu E, Soran M, Öz Ş (2024). The relationship between uropathogens and clinical characteristics of children with urinary tract infection. Health Sci Qtrly.

[12] Wieczorek M, Schwarz F, Sadlon A (2022). Iron deficiency and biomarkers of inflammation: a 3-year prospective analysis of the DO-HEALTH trial. Aging Clin Exp Res.

[13] Dimković N (2024). Uncomplicated urinary tract infections in the elderly. Galenika Med J.

[14] Zhang H, Wang X, Zhang Q, Xia Y, Liu D (2017). Comparison of procalcitonin and high-sensitivity C-reactive protein for the diagnosis of sepsis and septic shock in the oldest old patients. BMC Geriatr.

[15] Kang H (2021). Sample size determination and power analysis using the G*Power software. J Educ Eval Health Prof.

[16] Sabih A, Leslie SW (2025). Complicated Urinary Tract Infections. https://www.ncbi.nlm.nih.gov/books/NBK436013/.

[17] Mike-Ogburia M, Monsi T, Nwokah E (2023). Prevalence and associated risk factors of uropathogenic Klebsiella species in Port Harcourt. Adv Infect Dis.

[18] Hamid R, Losco G (2014). Pelvic organ prolapse-associated cystitis. Curr Bladder Dysfunct Rep.

[19] Karslıoğlu M, Yılmaz M (2024). Exploring the nexus: prevalence, risk factors, and clinical correlations of urinary tract infections in diabetes mellitus patients - a comprehensive retrospective analysis. Anatol Curr Med J.

[20] Pacurari A, Serban C, Narita A (2010). Is urinary tract infection a risk factor for hypertension in the elderly?. J Hypertens.

[21] Trye A, Krishnamoorthy K, Gittens‐Williams L (2022). Pyelonephritis in pregnancy. Top Obstet Gynecol.

[22] Choi JK, Kwon JC (2015). Bilateral psoas muscle abscess associated with emphysematous cystitis. Case Rep Med.

[23] Aleksić P, Bančević V, Aleksić A (2015). Antibiotic ointment in the management of recurrent, community-acquired bacterial cystitis in women: observational study. Med Istraz.

[24] Alaryan R, Alajmi A, Alkhudhair O (2022). Overview of the causes and types of recurrent cystitis. Int J Community Med Public Health.

[25] Manoppo M, Tendean H, Sondakh J (2017). High-sensitivity C-reactive protein (hsCRP) level on premature rupture of membrane (PROM) at term pregnancy. Indones J Obstet Gynecol.

[26] Song IU, Kim JS, Chung SW, Lee KS (2009). Is there an association between the level of high-sensitivity C-reactive protein and idiopathic Parkinson's disease? A comparison of Parkinson's disease patients, disease controls and healthy individuals. Eur Neurol.

[27] Benea A, Turaiche M, Rosca O (2025). Comparative assessment of lower urinary tract infections in hospitalized adults from western Romania: a retrospective cohort with microbiological analysis. Microorganisms.

[28] Chuang HH, Lin RH, Li WC, Yeh WC, Lin YA, Chen JY (2020). High-sensitivity C-reactive protein elevation is independently associated with subclinical renal impairment in the middle-aged and elderly population-a community-based study in northern Taiwan. Int J Environ Res Public Health.

[29] Nickavar A, Safaeian B, Valavi E (2016). Evaluation and comparison of urinary cytokines for the diagnosis of acute pyelonephritis. Arch Pediatr Infect Dis.

[30] Islam M (2024). Bacteriological profile and antimicrobial resistance patterns in pediatric urinary tract infections: a cross-sectional study at Dhaka Shishu Hospital. SSB Glob J Med Sci.

